# Supplementary material for: Systematic review the efficacy and safety of cilostazol, pentoxifylline, beraprost in the treatment of intermittent claudication: A network meta-analysis
Source: PLoS One. 2022 Nov 1;17(11):e0275392. doi: 10.1371/journal.pone.0275392 (PMC9624404; doi:10.1371/journal.pone.0275392)
Supplement: S1 Table — (DOCX) [file pone.0275392.s001.docx]

S1 Table Heterogeneity assessment of all pairwise comparisons for different outcomes

| outcomes | pairwise comparison | Higgins' I^2^ | P | N (study) |
| --- | --- | --- | --- | --- |
| MWD | placebo VS cilostazol | 49.4 | 0.118 | 12 |
|  | placebo VS pentoxifylline | 76.0 | 0.010 | 6 |
|  | placebo VS beraprost | 86.7 | 0.001 | 3 |
|  | cilostazol VS pentoxifylline | 38.2 | 0.167 | 6 |
| PFWD | placebo VS cilostazol | 42.7 | 0.148 | 8 |
|  | placebo VS pentoxifylline | 0.0 | 0.634 | 9 |
|  | placebo VS beraprost | 64.7 | 0.092 | 3 |
|  | cilostazol VS pentoxifylline | 10.6 | 0.290 | 2 |
| ABI | placebo VS cilostazol | 82.2 | 0.000 | 6 |
|  | placebo VS pentoxifylline | 0.0 | 0.454 | 2 |
|  | placebo VS beraprost | 85.5 | 0.009 | 2 |
| AE | placebo VS cilostazol | 0.0 | 0.452 | 12 |
|  | placebo VS pentoxifylline | 0.0 | 0.513 | 7 |
|  | placebo VS beraprost | 0.0 | 0.835 | 5 |
