# Supplementary material for: Systematic review the efficacy and safety of cilostazol, pentoxifylline, beraprost in the treatment of intermittent claudication: A network meta-analysis
Source: PLoS One. 2022 Nov 1;17(11):e0275392. doi: 10.1371/journal.pone.0275392 (PMC9624404; doi:10.1371/journal.pone.0275392)
Supplement: S2 Table — (DOCX) [file pone.0275392.s002.docx]

S2 Table The ranking probabilities in MWD

| ranking in MWD | placebo | cilostazol | pentoxifylline | beraprost |
| --- | --- | --- | --- | --- |
| Best | 0.0 | 78.8 | 0.6 | 20.5 |
| 2nd | 0.0 | 20.8 | 35.6 | 43.6 |
| 3rd | 2.3 | 0.4 | 63.7 | 33.6 |
| Worst | 97.7 | 0.0 | 0.0 | 2.2 |
