# Supplementary material for: Systematic review the efficacy and safety of cilostazol, pentoxifylline, beraprost in the treatment of intermittent claudication: A network meta-analysis
Source: PLoS One. 2022 Nov 1;17(11):e0275392. doi: 10.1371/journal.pone.0275392 (PMC9624404; doi:10.1371/journal.pone.0275392)
Supplement: S3 Table — (DOCX) [file pone.0275392.s003.docx]

S3 Table The ranking probabilities in PFWD

| ranking in PFWD | placebo | cilostazol | pentoxifylline | beraprost |
| --- | --- | --- | --- | --- |
| Best | 0.0 | 57.3 | 9.2 | 33.5 |
| 2nd | 0.0 | 33.7 | 38.6 | 27.7 |
| 3rd | 5.1 | 9 | 52 | 34 |
| Worst | 94.9 | 0.0 | 0.3 | 4.8 |
