# Supplementary material for: Systematic review the efficacy and safety of cilostazol, pentoxifylline, beraprost in the treatment of intermittent claudication: A network meta-analysis
Source: PLoS One. 2022 Nov 1;17(11):e0275392. doi: 10.1371/journal.pone.0275392 (PMC9624404; doi:10.1371/journal.pone.0275392)
Supplement: S4 Table — (DOCX) [file pone.0275392.s004.docx]

S4 Table The ranking probabilities in ABI

| ranking in ABI | placebo | cilostazol | pentoxifylline | beraprost | B + C |
| --- | --- | --- | --- | --- | --- |
| Best | 0 | 0 | 0 | 8.6 | 91.4 |
| 2nd | 0 | 0 | 0 | 91.4 | 8.6 |
| 3rd | 0 | 98.4 | 1.6 | 0 | 0 |
| 4th | 67.9 | 1.6 | 30.5 | 0 | 0 |
| Worst | 32.1 | 0 | 67.9 | 0 | 0 |
