# Supplementary material for: Systematic review the efficacy and safety of cilostazol, pentoxifylline, beraprost in the treatment of intermittent claudication: A network meta-analysis
Source: PLoS One. 2022 Nov 1;17(11):e0275392. doi: 10.1371/journal.pone.0275392 (PMC9624404; doi:10.1371/journal.pone.0275392)
Supplement: S5 Table — (DOCX) [file pone.0275392.s005.docx]

S5 Table The ranking probabilities in AE

| ranking in AE | placebo | cilostazol | pentoxifylline | beraprost | B + C |
| --- | --- | --- | --- | --- | --- |
| Best | 75.1 | 1.1 | 4 | 0 | 19.8 |
| 2nd | 23.1 | 30.1 | 36.2 | 1 | 9.6 |
| 3rd | 1.8 | 49.9 | 37.9 | 3.1 | 7.3 |
| 4th | 0.1 | 18.3 | 20.3 | 41.1 | 20.2 |
| Worst | 0 | 0.6 | 1.6 | 54.9 | 43 |
