# Supplementary material for: Systematic review the efficacy and safety of cilostazol, pentoxifylline, beraprost in the treatment of intermittent claudication: A network meta-analysis
Source: PLoS One. 2022 Nov 1;17(11):e0275392. doi: 10.1371/journal.pone.0275392 (PMC9624404; doi:10.1371/journal.pone.0275392)
Supplement: Attachment — Submitted filename: Response to Reviewers.docx [file pone.0275392.s008.docx]

Dear reviewer,

Thank you very much for your comments and professional advice. These opinions help to improve academic rigor of our article. Based on your suggestion and request, we have made corrected modifications on the revised manuscript.

Reviewer #2:

1. While the authors have corrected the spelling of “beraprost” throughout the manuscript, they have not made the appropriate change in the title of the study. This would be an essential edit as it would play a significant role in the visibility of the article in searches.

**Response: We have checked the manuscript and the title again, and corrected the** misspelling.
2. In line 266, the phrase “leaving clinicians know about a drug to prescribe”, is poorly phrased and may confuse readers. The authors should revise it appropriately to improve readability.

**Response: We have revised the phrase “leaving clinicians know about a drug to prescribe” as “providing guidance for clinicians in practice”.**

3. In line 300, the authors should consider omitting the word “therefore” from the sentence “Therefore, in addition to exploring the direct....”.

**Response: We have revised this sentence in line 300.**

Thank you very much for your attention and time. Look forward to hearing from you.
